# Community resilience among Ukrainian refugees: what is the role of the host community in recovery from forced migration?

**DOI:** 10.3389/fpsyt.2023.1206304

**Published:** 2023-08-03

**Authors:** Laura Migliorini, Martina Olcese, Paola Cardinali, Davide Prestia

**Affiliations:** ^1^Department of Educational Science, University of Genoa, Genoa, Italy; ^2^Department of Economics, Mercatorum University, Rome, Italy; ^3^IRCCS Ospedale Policlinico San Martino, Genoa, Italy

**Keywords:** community resilience, wellbeing, forced migration, Ukrainian refugees, trauma, stress disorders, protective factors

## Introduction

The Russian-Ukrainian conflict, which began in February 2022, has triggered a catastrophic and rapidly growing humanitarian emergency and displacement, threatening the stability of geopolitical relations. At the time of writing, the number of border crossings from Ukraine recorded since the beginning of the war stands at 19,729,989 and the number of Ukrainian refugees registered in Europe stands at 8,157,230, most of whom are women and children because men have been drafted into the armed forces since the conflict began (1, 2). Regarding the experience of forced migration, the literature highlights several traumatic factors that can compromise the mental health of these people (3, 4). Traumatic factors can be classified in relation to the period of forced migration. Before migration, people may experience violence, loss of family or community members, and disruption of family and community life; difficult and strenuous conditions may occur during the journey to move and, finally, reached host countries people can experience social isolation, joblessness and unemployment, language difficulties, different forms of persecution and acculturative stress (5, 6). These experiences are associated with psychological distress and an increased risk of psychiatric disorders; women are also more likely to develop internalizing symptoms (7). In addition, these people are at high risk of developing anxiety and depressive disorders, post-traumatic stress disorder (PTSD), and suicidal ideation/behavior (8–11). To reduce the risk of psychopathology and mental suffering and to improve recovery it is therefore necessary to promote different protective factors, not only at the individual level, but also at the community level (12, 13). It is therefore important to carry out community actions outside of psychiatric service delivery settings to facilitate prevention (14).

## Protective factors of community

To address these traumatic factors, from an ecological and multilevel perspective, the community, with its resources, can become a protective factor (15). Among other definitions, in the psychological context, community has been defined as a group of people who share the same values and interests and have similar experiences and needs (16). Concerning community protective factors in forced migration, it is possible to refer to both the migrant community and the host community. On one hand, different aspects of the migrant community can be relevant to cope with the difficulties of forced migration. In fact, faith in the cause of the community, a sense of pride in belonging to one's own ethnic community and the maintenance of one's own community values are protective aspects that facilitate the process of forced migration (6, 17). On the other hand, the host community with its attitude of acculturation can foster the process of hospitality of forced migrants by promoting multiculturalism and not considering migrants as competitors for material and intangible resources (18). In relation to these aspects the protective factor of resilience, understood as the ability to deal with traumatic events by finding the available resources, can also be analyzed at the community level and not only at individual level (19).

## Community resilience and related factors

Community resilience understood as the ability to recover from traumatic environmental, economic, or social events and to prepare for future adverse events is conceptualized as a set of adaptive capabilities that can be categorized into community competence, information and communications, economic aspects, and social capital and can be used as a conceptual framework to promote refugee integration and wellbeing and prevent the onset of psychiatric disorders (20–23). In order to reduce the risk of psychopathology of these people, it was deemed necessary to implement mental health and psychosocial support programs (24). Therefore, interventions to promote community resilience for refugees were seen as necessary by linking the community dimension with individual-focused interventions and treatment to promote mental and physical health (25). In line with this, implementing programs in the host country that can foster the different dimensions of community resilience is a relevant aspect (19). In fact, the literature highlights the interventions that promote community competence, such as the sense of agency and empowerment, a sense of cooperation and common vision by creating moments dedicated to the sharing of one's own history or culture through the organization of multicultural meetings, day trips, or celebrating holidays typical of the ethnic culture of belonging (26). Furthermore, the host community can facilitate community resilience by promoting the social support offered by the different formal and informal community services such as the church or voluntary associations and by encouraging the creation of social networks to reduce the risk of isolation (27). In addition, providing adequate information about the host culture and political rights of refugees, promoting the dissemination of positive narratives about migrants, and encouraging access to economic resources through the implementation of job training or economic benefits deriving from the assistance of local non-profit organizations, are key aspects of promoting community resilience (17, 28). Finally, to promote community resilience by enhancing the various factors that characterize the community, the host context might take action to strengthen social support networks, create collaborations and partnerships among community organizations, enhance community resources, and promote the building of a community identity based on cohesion and agency (16, 29–31).

### Promoting community resilience in Ukrainian refugees

The Inter-Agency Standing Committee (IASC) Guidelines (32) emphasize the importance of involving community members in coping with emergencies. Bhugra et al. (33) highlight in their recommendations to policymakers the importance of involving migrants in various actions such as cultural competence training. Finally, it is necessary to host Ukrainians, with their specificity of being predominantly refugee women and children who have often found refuge in countries neighboring Ukraine, where there is greater cultural proximity and possibility of return to their homeland, helping them to become citizens and to contribute their resources and talents to a cultural exchange with the host context (34, 35). In line with this to promote community resilience among forced migrants, one useful approach is community-based participatory research (CBPR) (36). CBPR is a research orientation that involves an equal partnership between communities and researchers, incorporating community theories, participation and practices into research efforts. This model fosters community health promotion through a participatory approach in terms of intervention design and implementation (37). This mechanism creates a process of empowerment through the sharing of information, resources, support and decision-making power (38).

The use of CBPR, with constant involvement of the migrant community and its representatives helps in tailoring services to the specific ethnic and cultural context of the community, avoiding the implementation of general actions that may not be well-suited for the population involved. This targeted approach is identified as key in the promotion of mental health and the prevention of mental disorders in humanitarian emergencies (39).

Promoting community resilience among Ukrainian migrants through a Community-Based Participatory Research (CBPR) method can be highly effective in addressing their unique needs and fostering empowerment as evidenced with refugees of other nationalities (40). Table 1 shows how CBPR can be applied, along with practical examples with Ukrainian community inspired by the key actions of the guidelines on mental health and on the emergencies and literature on Ukrainian refugees and CBPR approach (22, 32, 33, 41).

**Table 1:** Practical examples proposal of CBPR method's steps with the Ukrainian refugees.

**Identifying community needs and resources**	**Collaborative planning and decision-making**	**Capacity building and skill development**	**Action and evaluation**
- Engage Ukrainian migrants and community members as active participants in the research process to identify their specific needs and resources. - Gather information on the key issues affecting the community, such as language barriers, employment opportunities, or cultural adaptation challenges. But also, data related to the characteristics of refugees (gender, average age, level of schooling etc.) to design specific actions for in relation to these aspects. - Involving experts in Ukrainian culture, such as representatives of Ukrainian associations in a host context, promotes a targeted knowledge of culture and the possibility of planning actions aimed at Ukrainian ethnic cultural identity.	- Establish partnerships between Ukrainian migrants, community organizations, researchers, and relevant stakeholders to jointly develop strategies and interventions. - Collaborate with educational institutions, NGOs, and vocational training centers to provide access to education and training opportunities for Ukrainian refugee's children. - Hold community meetings to ensure that the voices of Ukrainian migrants are heard and that their priorities are reflected in the planning process.	- Provide training and capacity-building opportunities for Ukrainian migrants to enhance their skills and knowledge about host countries and resources. - Offer workshops on topics such as community competency, advocacy, or community organizing to empower Ukrainian migrants to actively participate in decision-making processes and engage with local institutions. - Create opportunities for cultural exchange between the host community and Ukrainian refugees.	- Implement the planned interventions, monitoring their progress and evaluating their effectiveness in collaboration with the Ukrainian refugee's community. - Continuously engage community members in the evaluation process, gathering feedback.
**Example:** Collaborate with a refugee hosting association to conduct focus groups of Ukrainian refugees, collecting data on their experiences in the host context and identifying areas where community resilience-building efforts are most needed relation to their ethnic cultural identity.	**Example:** Organize a community planning workshop where Ukrainian migrants, stakeholders, and researchers come together to co-design programs that address the identified needs, such as language classes, job placement initiatives, social support, and mental health services. Promoting moments of cultural exchange and promotion of their ethnic culture within the educational institutions where children may have been placed.	**Example:** Facilitate a series of workshops on community organizing and advocacy, equipping Ukrainian migrants with the skills and knowledge needed to effectively advocate for their rights and access resources within the host society. Furthermore, facilitating cultural exchange between the host community and Ukrainian refugees through the promotion of events highlighting Ukrainian culture related to, e.g., traditions, food or religion.	**Example:** Gathering feedback from refugees involved in the interventions on their experiences and the benefits gained.

## Discussion

In this opinion article, we presented the community resilience approach to promoting refugees' integration and wellbeing and preventing the onset of psychiatric disorders, which could also be applied to Ukrainian migrants. In our opinion hosting refugees is a challenge also for the host community, which is called upon to deploy tangible and intangible resources that can promote community resilience and reduce risk factors for the development of mental health problems and psychiatric disorders among refugees, with a consequent reduction of health care costs. At the same time, a resilient refugee community can increase the resources of the host country. Indeed, in line with this framework, it can be argued that the design and implementation of community-centered interventions, with a focus on community resilience, are key aspects to protect the traumatic factors to which these people are exposed.

Given the difficult situation of Ukrainian immigrants, host countries might take steps to promote projects and actions that can foster the welfare of the individual at the community level, in addition to the individual. Such projects should strength the resilience of the refugee community who are facing the trauma of war and forced migration and who may later face a possible return to their country with its attendant difficulties. In order to do that, it is necessary to implement projects in which the community is involved, in which it's possible to promote agency and empowerment, and in which the different resilience factors of the community can be fostered, in a collaborative approach between political institutions and various community actors, e.g., through the CBPR methodology that involves community members in the whole process and promotes the implementation of actions related to the ethnic, cultural and sociodemographic specificity of Ukrainian refugees. Indeed, CBPR methodology can empowers Ukrainian migrant communities to actively participate in research and intervention processes, fostering community resilience. By leveraging their strengths and collaborating closely with community members, researchers can create impactful initiatives tailored to the specific needs of Ukrainian migrants.

In conclusion, further works are needed to clarify effective ways to implement projects by host countries to empower the community resilience of Ukrainian refugees.

## Author contributions

LM and DP developed the idea presented. PC and MO developed the theory and drafted the manuscript. All authors critically reviewed the manuscript and gave their final approval.
